# epiXact-ONT: Long-read whole genome sequencing for rapid outbreak detection and comprehensive plasmid transmission analysis

**DOI:** 10.1017/ash.2024.335

**Published:** 2024-09-16

**Authors:** Emma Briars, Ian Herriott, Allison Brookhart, Talia Hollowell, Nicole Billings, Miriam Huntley, Mohamad Sater

**Affiliations:** Day Zero Diagnostics

## Abstract

**Background:** Healthcare associated infections (HAIs) are a major contributor to patient morbidity and mortality. HAIs are increasingly important due to the rise of multidrug resistant pathogens which can lead to deadly nosocomial outbreaks. Traditional methods for investigating transmissions are slow, costly and have poor detection resolution. In addition, plasmid transmission which can horizontally transfer critical resistance and virulence genes is not part of routine infection control practice due to lack of comprehensive and cost-effective methods capable of identifying both pathogen and/or plasmid transmission. Here we demonstrate the utility of the Oxford Nanopore Technologies (ONT) platform for whole genome sequencing (WGS) based pathogen and plasmids transmission analysis. **Methods:** We developed a rapid end-to-end process that includes sample preparation, sequencing optimized for generating long-reads and bioinformatics workflow customized for error-prone ONT data. We use Flye to generate de novo assemblies and a secondary bioinformatics step to identify each circular sequence. Individual circular sequences with an Ori (≥1) are identified. For pathogen clonality analysis we perform a pairwise mapping-based chromosomal sequences comparison eliminating need for an external reference genome. Similarly, individual plasmids are separated and compared pairwise. We annotate both the circularized chromosomal and plasmid sequences for known resistance and virulence genes. **Results:** We performed ONT (and confirmatory Illumina) sequencing of the genomes of 20 bacterial isolates originating from 5 HAI investigations previously performed at Day Zero Diagnostics using epiXact®, our Illumina-based HAI sequencing and analysis lab service. ONT-based clonality determination had 100% agreement with the Illumina based pipeline. We also found that using the outbreak-specific assembled genomes instead of an external reference increased the SNP-calling resolution in the ONT pipeline. We also identified sets of clonal isolates with both identical plasmids and distinct plasmids; as well as sets of non-clonal isolates with identical plasmids and distinct plasmids. In one subset of 7 multi-species isolates, we identified 2-7 circularized plasmid sequences in each isolate, all harboring known resistance genes. 4 plasmids were found in multiple isolates, with each plasmid appearing in between 2 and 4 distinct isolates. Notably, blaNDM was identified in at least 1 plasmid in each isolate. **Conclusion:** We demonstrate the utility of ONT for comprehensive HAI investigations, establishing the potential to transform healthcare epidemiology with rapid outbreak determination covering pathogen and plasmid transmission in < 2 4 hours from sample receipt.

